# Book Review

**Published:** 1949-10

**Authors:** 


### Reviews of Books

**Psychological Aspects of Clinical Medicine.** By S. B. HALL, M.D., D.P.M. Pp. xii, 416. Illustrated. London: H. K. Lewis & Co. Ltd. 1949. Price 21s.—This book states in the Introduction that it is based upon a course of clinical lectures for final-year and post-graduate students at the Liverpool Medical School: such students were very fortunate both in their lecturer and in his method of presentation of his subject. He gives a considerable amount of space to a general discussion of mental disease, but his whole object is to point out that man is " one and indivisible " and that no form of disorder can be assigned to a watertight compartment of any one particular system. He shows how even diseases which are obviously organic may be profoundly influenced by the psychological aspect and how also, in turn, such diseases may influence the patient's mind; in fact, he takes the reader rapidly but efficiently through a very large number of conditions in which he points out this interrelationship and shows how such knowledge may be utilized. It is particularly admirable to see the way in which he presents all sides of questions in a small space, and does not commit himself to any one particular school of thought in his approach to psychological problems to the exclusion of all others. We recommend this book with confidence.

**Surgery of the Colon and Rectum.** By Sir HUGH DEVINE, M.S., F.R.C.S., F.R.A.C.S., F.A.C.S., and JOHN DEVINE, M.S., F.R.C.S., F.R.A.C.S., F.A.C.S. Pp. xi, 362. Illustrated. Bristol: John Wright & Sons Ltd. 1948. Price 52s. 6d.—The name of Devine has for long been associated with the surgery of the colon and rectum, and this book can be regarded as representing the life-work of one who has devoted himself almost exclusively to the surgical problems of the large bowel. This is essentially a practical book: eminently readable and written in plain, straightforward English, it should appeal alike to the experienced surgeon and to those who are about to start on the long road to surgical efficiency. As a ready-reference book it should be invaluable, for the surgical matter is well arranged and tabulated. The earlier chapters deal comprehensively with the various benign and malignant lesions of the colon and rectum, but the theme running throughout the section on the various operative procedures is the authors' obvious preference for the multiple-stage and exteriorization methods of resection. This is particularly interesting at the present time, when the general trend may be fairly described as favouring rather the segmental resection with primary anastomosis, than the older, more established methods of Mikulicz and Paul. With the many chemotherapeutic agents now available there is much to be said for primary resection and anastomosis in a properly prepared case. The authors admit, however, that the extended extraperitoneal operation is not always applicable or desirable, and there are occasions when partial colectomy with primary suture must be employed. The book is well illustrated with numerous plates, some in colour. It is neat and compact in size, can be handled easily, is beautifully produced and can be thoroughly recommended.

**Blood Transfusion.** Edited by GEOFFREY KEYNES, M.D., F.R.C.S. Pp. xii, 574. Illustrated. Bristol: John Wright & Sons Ltd. 1949. Price 52s. 6d.—The aim of this book is to represent the state of knowledge and the essentials in the practice of blood transfusion at the present time. It has been compiled by a number of contributors, each having special experience in a particular aspect of transfusion work, clinical, laboratory and organizing. The book is in ten sections and covers the indications, complications and technique of transfusion in adults and in children, blood grouping, storage and drying, blood substitutes, donor aspects and the organization of a hospital transfusion department. Treatment by the transfusion of blood, its derivatives and substitutes, has so progressed during the past ten years that a comprehensive textbook dealing with all aspects of transfusion work has become highly desirable. The indications and complications of transfusion are fully described in two sections by Dr. Bodley Scott. Transfusion is represented as a replacement therapy, a view with which the majority of clinicians will be in agreement. These sections almost certainly contain too much detail for the non-specialist reader, however, and they might be improved by presenting the more detailed information in small type. Because of this attention to detail there is a tendency to lose sight of the transfusion essentials. This is particularly noticeable in the paragraphs dealing with the thrombocytopenic purpuras.

Clinical pathologists will find the blood-group section valuable and comprehensive, but, again, this section would provide clearer guidance to the less experienced if the paragraphs referring to underlying principles were clearly separated from those describing technical procedures. It would seem that this book's greatest value will be as a work of reference rather than as a guide to everyday transfusion practice for students and recently qualified practitioners. It is too detailed and insufficiently concise for the latter. It has two outstanding defects. The majority of contributors have not confined themselves to that particular aspect upon which they can claim expert knowledge, but have trespassed upon the subject-matter of some of their fellow contributors. In consequence, not only is space taken up by unnecessary repetition, but the reader will find himself offered contradictory advice upon a number of important points. The second major defect lies in the fact that insufficient attention has been given to the detailed organization of a blood-transfusion service other than in a large metropolitan hospital. Those concerned with transfusion work are faced with two main problems ; to ensure that a safe, compatible transfusion fluid is made available for all patients requiring transfusion therapy when and where it is needed, sufficient in amount and accompanied by a suitable giving apparatus ; and to see that the indications and hazards of this type of therapy are widely known, so that the fluids available are used to the patient's best advantage. Mr. Geoffrey Keynes's book should help to make transfusion safer, but it gives little guidance upon the other important problem of ensuring that more lives will be saved by transfusion outside the larger and fully-equipped hospitals.

**Neurological Anatomy.** By A. BRODAL. Pp. xv, 496. Illustrated. Oxford : Clarendon Press. 1948. Price 42s.—The already thin dividing line between neuroanatomy and neurophysiology has been even more narrowed by Professor Brodal's book. This book is invaluable for the clinician, and both neurologists and neurosurgeons will benefit by the care which is given to the link between neuroanatomy and disease of the nervous system. Particularly valuable is the clear summary of the work of Weddell and others in electromyography and its clinical application, and work on the somatic afferent system which emphasizes the growing difficulties in the acceptance of Head's original theories. In his remarks in the examination of the sensory system, Professor Brodal emphasizes that the examination should be conducted in stages and neither the patient nor the examiner must be fatigued. It is a lesson which quite a number of neurologists might well take to heart, as one still sees sensory examinations being pursued for a matter of hours, which inevitably leads to invalid results due to fatigue. This book is the most useful and complete summary we have of the work of anatomists and physiologists in the nervous system during the war years, and Professor Brodal is to be congratulated on his work.

**Massage and Remedial Exercises.** By NOEL M. TIDY. Eighth Edition. Pp. viii, 487. Illustrated. Bristol : John Wright & Sons Ltd. 1949. Price 25s.—Although it is only about two years since we reviewed the seventh edition of Tidy, there are a few additions and improvements to note. Sciatica due to prolapsed intervertebral disc is neatly explained and instructions given for remedial exercises, whether laminectomy is performed or not. Prenatal exercises have a place and physical treatment of asthma has been revised, as have anterior poliomyelitis and œdema after fractures. War gas-lesions have been omitted, so that this edition is only seven pages longer than the seventh.

**Cunningham's Manual of Practical Anatomy.** 3 Vols. Edited by J. C. BRASH, M.C., M.D., F.R.C.S., F.R.S.E. Eleventh Edition. Pp. xix, 387; x, 488; x, 513. Illustrated. London: Oxford University Press. (Geoffrey Cumberlege). 1948. Price 21s. per vol.—The Eleventh Edition of *Cunningham's Manual of Practical Anatomy* has been revised and edited by Professor J. C. Brash alone. The typography and illustrations are excellent, but in view of the frequent—and at times not too gentle—usage which the book receives in the dissecting-room, a more robust binding would appear to be indicated. The style is singularly well adapted to the needs of the subject, though one could hardly expect a manual of dissection to be a literary effort of the first order. Of particular interest are the two paragraphs in the general introduction, on the "Anatomy of the Living Body". It is probably safe to predict that in future editions of anatomical text-books the subject of living anatomy will be given an increasing proportion of the available space.

